# An Adult Case of Chest Wall Langerhans Cell Histiocytosis Mimicking Malignancy and Responding to Targeted Therapy

**DOI:** 10.7759/cureus.100949

**Published:** 2026-01-06

**Authors:** Michael W Alchaer, Trina Capelli, Thomas A Abbruzzese, Alene Wright

**Affiliations:** 1 General Surgery, HCA Healthcare/University of South Florida (USF) Morsani College of Medicine Graduate Medical Education, HCA Florida Brandon Hospital, Brandon, USA

**Keywords:** adult case, chest wall mass, langerhans cell histiocytosis, raf v600e mutation, targeted therapy

## Abstract

Langerhans cell histiocytosis (LCH) is a rare clonal proliferation of dendritic cells that is exceptionally uncommon in adults and only rarely affects the chest wall. Adult rib lesions often radiographically mimic malignancy, necessitating biopsy for accurate diagnosis.

We report the case of a 52-year-old woman with Class III (severe) obesity and newly diagnosed type 2 diabetes who developed a painful right chest wall mass following severe coughing during coronavirus disease 2019 (COVID-19). Imaging revealed a lobulated lesion with rib erosion and pleural indentation concerning for malignancy. Surgical debridement yielded histiocytic inflammation, and cultures grew Staphylococcus epidermidis. Final histopathology confirmed LCH. Despite wound re-closures and antibiotic therapy, persistent drainage continued until molecular testing identified a B-Raf proto-oncogene, serine/threonine-protein kinase (BRAF) V600E mutation. Targeted therapy with dabrafenib and trametinib was initiated, resulting in rapid clinical improvement and complete wound healing.

Adult chest wall LCH represents a rare diagnostic challenge due to its malignant radiographic appearance. Histopathologic confirmation, with molecular testing, is essential, and targeted BRAF/mitogen-activated protein kinase kinase (MEK) inhibition may provide an effective therapeutic option when conventional measures fail.

## Introduction

Langerhans cell histiocytosis (LCH) is a clonal proliferation of myeloid dendritic cells, rare in adults with an estimated incidence of 1-2 per million [1,2]. Chest wall involvement, such as rib or sternal lesions, is exceedingly uncommon and frequently misinterpreted as bone malignancy or infection [3-5].

Radiographically, LCH may mimic neoplastic processes due to findings such as rib erosion and soft-tissue extension, underscoring the importance of obtaining tissue diagnosis [6]. Molecular profiling has revealed activating mutations in the mitogen-activated protein kinase (MAPK) pathway, particularly the B-Raf proto-oncogene, serine/threonine-protein kinase (BRAF) V600E mutation, in approximately 40%-60% of adult LCH cases. This has enabled the use of targeted BRAF/mitogen-activated protein kinase kinase (MEK) inhibitors [7,8].

We present an adult case of chest wall LCH mimicking malignancy, complicated by refractory wound drainage, and successfully treated with dabrafenib and trametinib.

## Case presentation

A 52-year-old woman with Class III (severe) obesity; body mass index (BMI) 41.6, hypertension, and newly diagnosed type 2 diabetes presented with a painful right chest wall mass that developed following severe bouts of coughing during coronavirus disease 2019 (COVID-19) approximately five months prior to presentation [9]. Initially suspected to represent a rib fracture, the lesion progressively enlarged, prompting further evaluation.

**Figure 1:**
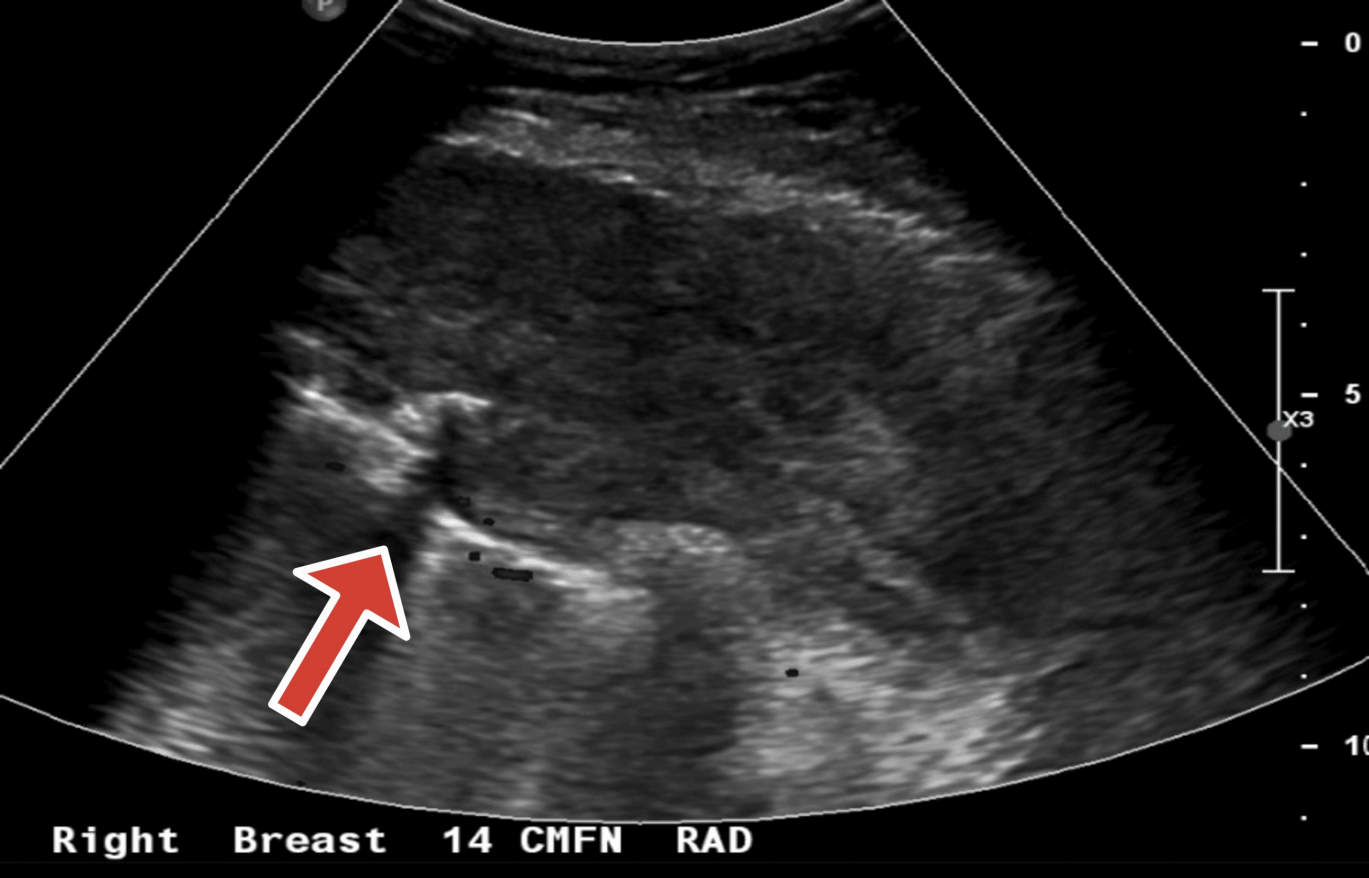
Ultrasound showing a heterogenous mass in the right anterior chest wall, extending to the subpleural space Arrow pointing at heterogeneous mass

**Figure 2:**
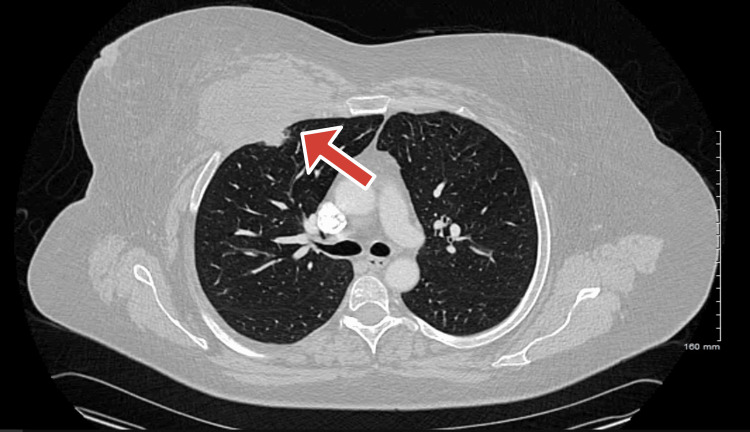
Chest CT with lobulated mass eroding the T3 rib and indenting the pleura, with extension between T3 and T6 ribs and an associated 3.3 × 2.1 cm anterior mediastinal lesion Arrow pointing at lobulated mass CT, computed tomography

Mammography revealed fatty-replaced breasts with focal asymmetry, calcifications, and axillary lymph nodes. Ultrasound demonstrated a heterogeneous 9.1 × 0.5 cm mass in the right anterior chest wall extending to the subpleural space (Figure 1). Chest computed tomography (CT) showed a 9.7 × 6.4 × 10.2 cm lobulated mass eroding the T3 rib and indenting the pleura, with extension between the T3-T6 ribs and an associated 3.3 × 2.1 cm anterior mediastinal lesion; no lymphadenopathy was identified (Figure 2).

Two months later, incision and drainage of a retropectoral abscess, debridement (4 × 5 × 6 cm), and sinus tract excision were performed. Preliminary pathology showed histiocytes, acute and chronic inflammation, multinucleated giant cells, necrosis, and granulation tissue. Cultures grew *Staphylococcus epidermidis*; fungal and acid-fast bacilli (AFB) cultures were negative. A 10-day course of levofloxacin was administered, resulting in initial improvement.

Final pathology (one month after the procedure) confirmed LCH with histiocytes extending to the surgical margins. Immunohistochemical staining demonstrated positivity for cluster of differentiation 1a (CD1a), S100 protein (S100), and langerin (CD207), consistent with LCH. Molecular testing identified a BRAF V600E mutation using polymerase chain reaction-based analysis.

Despite multiple attempts at wound closure and a course of trimethoprim-sulfamethoxazole, drainage and bleeding persisted. Approximately two months later, the patient commenced targeted therapy with dabrafenib and trametinib, resulting in complete wound resolution. At the three-month follow-up, she was asymptomatic with minimal residual skin changes.

## Discussion

Adult chest wall LCH is exceedingly rare, with only a handful of rib or sternal lesions reported in the literature [3-5]. The scarcity of documented cases makes clinical suspicion challenging, particularly when imaging reveals aggressive features such as cortical destruction and pleural invasion that can mimic malignancy and delay diagnosis [4].

Approximately 40%-60% of adult LCH cases harbor the BRAF V600E mutation, which drives disease pathogenesis and enables the use of molecularly targeted therapy [7,8,10]. Early pediatric trials of dabrafenib, alone or in combination with trametinib, demonstrated high response rates and durable remissions, and subsequent adult case series have reported similarly favorable outcomes [11-13]. The recent tissue-agnostic approval by the U.S. Food and Drug Administration (FDA) of dabrafenib and trametinib for BRAF V600E-mutated solid tumors further supports their role in histiocytic neoplasms [14].

In the present case, impaired wound healing and persistent drainage were likely exacerbated by diabetes and local infection, both well-recognized barriers to recovery in hyperglycemic patients. Chronic hyperglycemia is associated with macrophage dysfunction, impaired neutrophil chemotaxis and phagocytosis, reduced angiogenesis, and delayed granulation tissue formation, all of which contribute to impaired wound healing and increased susceptibility to infection [15,16]. Durable lesion resolution occurred only after systemic disease control with combined BRAF/MEK inhibition, underscoring the importance of addressing underlying disease activity when conventional surgical or antibiotic interventions fail [12,13].

This case emphasizes that LCH should remain in the differential diagnosis for destructive chest wall lesions in adults. Early histopathologic confirmation and molecular profiling are essential to guide effective management, avoid unnecessary repeat operations, and improve overall outcomes [3].

## Conclusions

This case emphasizes that adult chest wall masses with aggressive radiographic features may represent LCH rather than malignancy. Accurate diagnosis requires biopsy and molecular testing. In BRAF V600E-positive disease, targeted therapy with dabrafenib and trametinib can provide rapid, durable control, even when surgical interventions are complicated by infection and impaired healing. Incorporating LCH into the differential diagnosis of chest wall tumors is critical to avoid misdiagnosis and to guide patients toward effective, precision-based treatment.
